# Editorial: Hereditary colorectal cancer syndromes and risk assessment in the era of precision medicine

**DOI:** 10.3389/fonc.2024.1474157

**Published:** 2024-10-10

**Authors:** Kevin McDonnell, Gad Rennert, Gregory E. Idos

**Affiliations:** ^1^ Department of Medicine, City of Hope National Medical Center, Duarte, CA, United States; ^2^ Technion Israel Institute of Technology, Technion Israel Institute of Technology, Haifa, Israel

**Keywords:** precision medicine, hereditary, colorectal cancer, genetic testing, chemoprevention

Colorectal cancer (CRC) is a significant public health challenge, necessitating ongoing research into more effective diagnostic and treatment approaches. The Research Topic, “Hereditary Colorectal Cancer Syndromes and Risk Assessment in the Era of Precision Medicine,” explores and showcases the latest advancements in hereditary colorectal cancer (CRC) syndrome diagnosis, treatment, and management, providing a comprehensive overview of how precision medicine is transforming CRC care for individuals with genetic predispositions. The articles in this Research Topic offer cutting-edge insights into various aspects of CRC, from novel diagnostic tools to chemoprevention strategies, underlining the importance of precision medicine in enhancing patient outcomes.

Dong et al. present a novel approach to preoperative lymph node (LN) metastasis evaluation in rectal cancer. By integrating MRI radiomics with clinical data, they developed a machine learning model that surpasses traditional assessment methods in predicting LN metastasis, achieving a higher positive predictive value. The findings suggest that this approach could significantly aid in tailoring more precise treatment strategies, emphasizing the clinical utility of combining radiomics with conventional clinical data. This research exemplifies how precision medicine can enhance non-invasive diagnostic accuracy and assist in tailoring treatment strategies, thus improving management for patients with colorectal cancer.

Building upon this theme of innovative diagnostic techniques, O’Shea et al. introduce an advancement in integrating genomic testing into routine oncology practice. Their study presents a model, derived from comprehensive implementation research, provides a structured approach to improve access to genomic testing for Lynch syndrome. By addressing barriers such as funding, infrastructure, and role delineation, and proposing solutions like embedded genetic counselors and electronic medical record integration, this model aims to streamline genetic testing processes. Its successful implementation could serve as a blueprint for other hereditary cancer syndromes, facilitating broader adoption of precision medicine practices. This study aims to integrate precision medicine into routine oncology practice, ensuring that individuals with hereditary risks receive timely and accurate genetic testing and subsequent personalized care.

Complementing these advancements in diagnostics and genetic testing, Brand et al. explore the potential of cytokine profiling to predict colorectal cancer risk in Lynch syndrome patients. By analyzing normal-appearing colorectal tissue, the study identifies immune signatures that distinguish LS patients with a history of CRC from those without. These findings suggest that immune microenvironment profiling could become a valuable tool in CRC risk assessment and prevention strategies, enabling more personalized surveillance and intervention approaches.

While Brand et al. focus on risk prediction, another crucial aspect of personalized care for hereditary colorectal cancer syndromes is chemoprevention. In this vein, Mraz et al. provide valuable insights into how chemoprevention is widely discussed and applied, particularly in Lynch syndrome (LS) and familial adenomatous polyposis (FAP). There remains considerable variability in the specifics of these practices. Most professionals recommend aspirin for LS patients, albeit with no consensus on the optimal dosage. This heterogeneity underscores the need for standardized guidelines and further research to optimize chemoprevention strategies, considering individual patient factors to maximize benefits and minimize risks.

As the field of chemoprevention continues to evolve, so too does our understanding of genetic risk factors. Highlighting this progression, Li et al. demonstrate the critical role of genetic testing in hereditary cancer syndromes through their identification of a novel splicing variant in the MSH2 gene. This study not only confirms the pathogenicity of the MSH2 c.793-1G>A variant through bioinformatics and functional assays but also underscores the importance of regular surveillance for mutation carriers. Such genetic insights are vital for early detection and prevention of CRC in at-risk populations. By confirming the pathogenicity of this variant, this study supports the role of precision medicine in identifying genetic mutations and highlights the need for regular surveillance and personalized risk assessment for mutation carriers.

Moving from genetic risk factors to the broader molecular landscape of colorectal cancer, Luo et al. offer new perspectives on the molecular underpinnings of colon cancer through their exploration of cuproptosis-related genes. By identifying distinct molecular subtypes associated with different tumor microenvironment characteristics and treatment responses, their research contributes to the development of more tailored therapeutic strategies. This study exemplifies how precision medicine can guide the development of personalized treatment options for patients with hereditary predispositions to colon cancer, further emphasizing the importance of molecular profiling in advancing CRC care.

These studies collectively underscore the rapidly evolving landscape of CRC diagnosis and treatment, driven by advancements in genomics, radiomics, and immunology. By presenting these diverse contributions, we aim to provide a comprehensive overview of how precision medicine is reshaping our approach to hereditary CRC syndromes. Looking ahead, it will be crucial to continue validating these findings through large-scale, multicenter studies and to develop standardized guidelines for their clinical implementation. The integration of these innovative approaches holds promise for significantly improving patient outcomes and reducing the burden of colorectal cancer globally. By embracing the principles of precision medicine, healthcare providers can offer more targeted and effective care, ultimately transforming the management of colorectal cancer and other hereditary cancer syndromes.

